# Radiation therapy for ventricular arrhythmias

**DOI:** 10.1111/1754-9485.13662

**Published:** 2024-05-02

**Authors:** Xingzhou Liulu, Poornima Balaji, Jeffrey Barber, Kasun De Silva, Tiarne Murray, Andrew Hickey, Timothy Campbell, Jill Harris, Harriet Gee, Verity Ahern, Saurabh Kumar, Eric Hau, Pierre C Qian

**Affiliations:** ^1^ Cardiology Department Royal North Shore Hospital Sydney New South Wales Australia; ^2^ Cardiology Department, Westmead Hospital University of Sydney Sydney New South Wales Australia; ^3^ Westmead Applied Research Centre, Faculty of Medicine and Health University of Sydney Sydney New South Wales Australia; ^4^ Department of Radiation Oncology, Crown Princess Mary Cancer Centre Westmead Hospital Sydney New South Wales Australia; ^5^ Sydney Medical School, University of Sydney Sydney New South Wales Australia; ^6^ Translational Radiation Biology and Oncology Laboratory, Centre for Cancer Research The Westmead Institute for Medical Research Sydney New South Wales Australia; ^7^ Blacktown Hematology and Cancer Centre, Blacktown Hospital Blacktown New South Wales Australia

**Keywords:** ablation, non‐invasive, radiosurgery, stereotactic body radiation therapy, ventricular arrhythmias

## Abstract

Ventricular arrhythmias (VA) can be life‐threatening arrhythmias that result in significant morbidity and mortality. Catheter ablation (CA) is an invasive treatment modality that can be effective in the treatment of VA where medications fail. Recurrence occurs commonly following CA due to an inability to deliver lesions of adequate depth to cauterise the electrical circuits that drive VA or reach areas of scar responsible for VA. Stereotactic body radiotherapy is a non‐invasive treatment modality that allows volumetric delivery of energy to treat circuits that cannot be reached by CA. It overcomes the weaknesses of CA and has been successfully utilised in small clinical trials to treat refractory VA. This article summarises the current evidence for this novel treatment modality and the steps that will be required to bring it to the forefront of VA treatment.

## Introduction

Ventricular arrhythmia (VA) is a life‐threatening condition whereby frequent or continuous beats from the bottom chamber of the heart can cause syncope, heart failure or death. Implantable cardioverter defibrillators (ICD) save lives by terminating VA but do not modify the underlying mechanisms that drive this arrhythmia. Recurrent ICD shocks are associated with decreased quality of life and increased mortality.^1^, ^2^, ^3^ VA results from two different mechanisms: abnormal focal activity leading to automaticity or triggered activity, and re‐entry circuits.^4^ Abnormal focal activity results from membrane instability in poorly coupled myocytes and Purkinje cells.^5^ Re‐entry results from small bridges of viable myocytes within scar that are capable of sustaining continuous electrical circuits.^5^ Re‐entry is the predominant mechanism that causes VA in structural heart disease.^6^

**Fig. 1:**
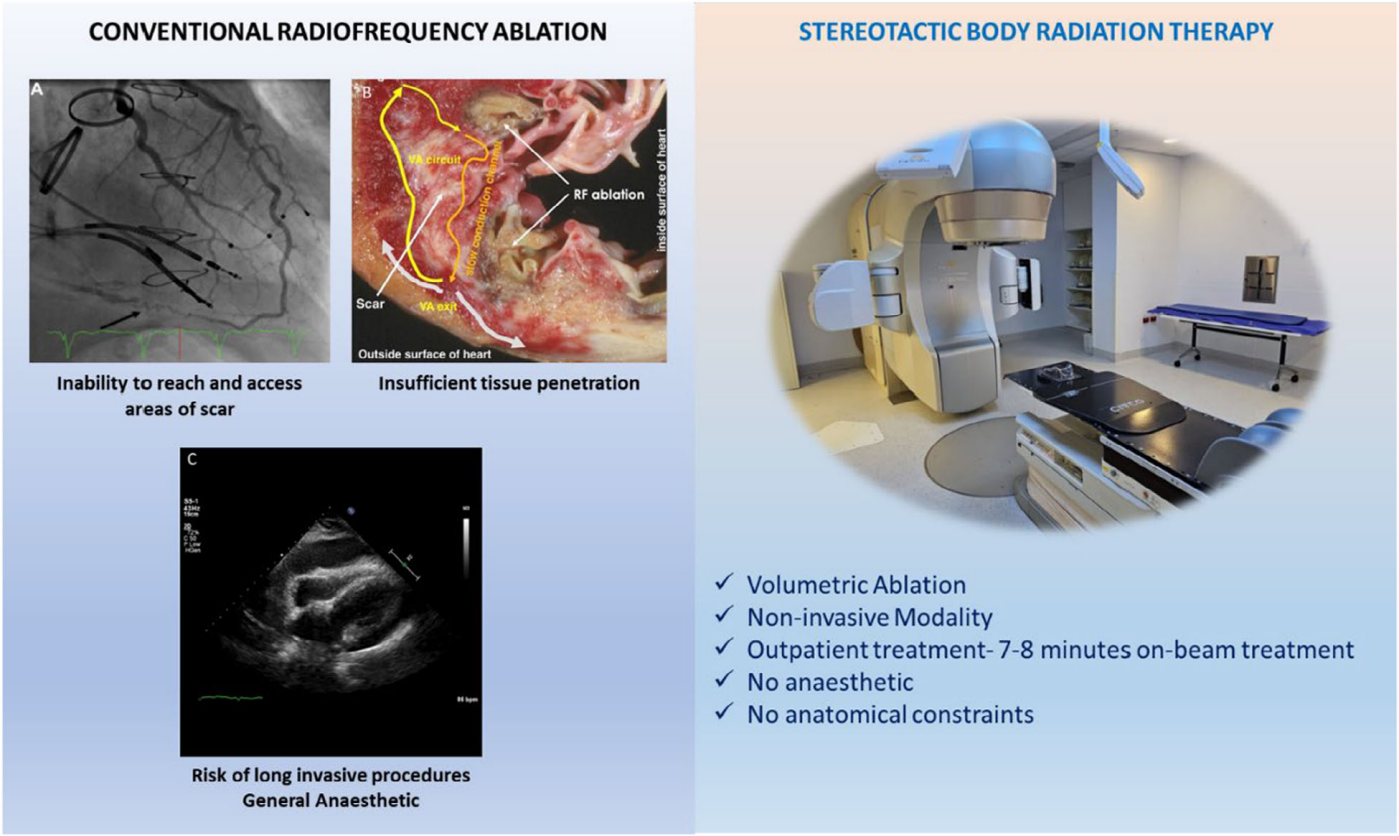
Weaknesses of conventional radiofrequency ablation compared to strengths of stereotactic radiotherapy. (a) Double mechanical valve limiting catheter access into left ventricle. (b) Inability of RF ablation to achieve ablation through scar. (c) Cardiac tamponade as a rare life‐threatening complication from CA. Reprinted [adapted] from Divakara *et al*.^128^

Catheter ablation (CA) with radiofrequency energy (RF) is the subsequent line of treatment when medications fail in VA. CA aims to find and cauterise viable myocytes within scar that form re‐entry circuits. Although CA has been demonstrated to be more effective than medication to control VA, the current success rate remains modest ranging from 50% to 70% in contemporary studies, with an overall major complication rate including death of 8.8%.^7^, ^8^, ^9^, ^10^ Treatment failure is due to several different factors (Fig. 1). Fibrosis and fat that develops within scar tissue shields deep underlying circuits that drive VA from effective eradication with RF energy.^11^ The area of scar may be inaccessible to catheters due to anatomical constraints such as mechanical valves.^12^ Alternative methods have been developed to improve lesion depth with RF such as combined epicardial ablation, use of bipolar ablation and novel ablation catheters such as the retractable needle tip electrode catheter.^13^, ^14^, ^15^ Neuromodulation of the sympathetic system through stellate ganglion block and bilateral cardiac sympathetic denervation has been utilised to successfully reduce VT burden.^16^, ^17^ There has been significant interest in different energy sources to treat refractory VT such as ethanol ablation, cryoablation and more recently electroporation or pulsed field ablation which has shown promise in achieving deeper lesions compared to RF.^18^, ^19^

Another alternative energy source is stereotactic body radiation therapy (SBRT), a mainstay treatment in oncology has now been adopted for use in the myocardium whereby clinicians can deliver radiation deep within myocardial tissue. Notably, it is a non‐invasive treatment modality, and there are promising early data from non‐randomised retrospective and prospective trials that it can treat VA in patients with advanced structural heart disease who are not amenable to further CA attempts. However, there are major gaps in knowledge regarding radiation dosing and delivery, long‐term adverse effects of SBRT and the underlying mechanisms that lead to VA suppression that need to be answered.

## Antiarrhythmic effect of cardiac SBRT

### Preclinical studies

Stereotactic body radiotherapy (SBRT) utilises the convergence of multiple intersecting beams of radiation to deliver high doses of ablative radiotherapy with sub‐millimetre precision whilst minimising damage to surrounding tissue through the sharp dose gradient it achieves.^20^ In 2010, Sharma *et al*. published a seminal preclinical study investigating the use of SBRT in a porcine model.^21^ They utilised the CyberKnife platform to deliver SBRT to the atrioventricular node, cavotricuspid isthmus and pulmonary veins.^21^ Doses of 25 Gy or higher were able to produce lesions similar to ablation in each of these areas. Histological analysis at 90 days following the treatment revealed well‐circumscribed, transmural lesions with loss of myocyte architecture and increased fibrin with minimal collateral damage.^21^ Subsequent studies have confirmed dose dependency with increasing fibrosis and earlier conduction block with increasing dosage.^22^, ^23^ These studies found that 25 Gy was the minimum dosage required to achieve myocardial fibrosis and conduction block in normal atrial tissue.^21^, ^22^, ^23^ Hence, 25 Gy has since been employed in subsequent clinical trials; however, the optimal dosage required to achieve VA suppression in myocardial scar remains unknown.

## Clinical outcomes

### Clinical studies

Building on the initial case report data,^24^ Cuculich *et al*. published a case series of five patients treated with SBRT.^25^ Median treatment time was 12 min. A marked 99.9% reduction in VA burden was observed after a 6‐week blanking period at a median of follow‐up of 12 months. 2 patients had recurrence of VA during follow‐up at 4 weeks and 9 months, respectively. One patient passed away 3 weeks post‐treatment from a fatal stroke; however, this patient had a history of atrial fibrillation and was not anticoagulated due to bleeding risk. Therefore, it is unclear whether SBRT contributed to this patient's death. Post‐mortem 3 weeks after treatment showed no acute myocardial inflammation, acute cellular necrosis or fibrosis.

A prospective single‐centre trial with 19 patients was published by Robinson *et al*., following this case series.^4^ 17 patients had refractory VA, and 2 patients had pre‐ventricular ectopic (PVC)‐induced cardiomyopathy. Median treatment time was 15.3 min. At 24 months, this cohort demonstrated a reduction to a median of 2 VA episodes and a persistent reduction of VA episodes in 16 out of 18 patients.^26^ An early reduction in VA episodes was noted in the 6‐week blanking period. At 3 months, there was 1 case of pericarditis which was conservatively managed and 1 exacerbation of heart failure that resulted in hospitalisation. Late side effects (>2 years) included a pericardial effusion which was medically managed and gastropericardial fistula requiring surgical repair. At 5 years, 15 of the 19 (79%) patients had passed away and freedom from any VT was 13%.^27^ Most deaths were due to cardiac failure (38%). Whilst this may represent the natural history of cardiomyopathy patients, whether SBRT accelerated heart failure progression in these patients is unknown.

To date, clinical outcome data for SBRT of cardiac arrhythmias continue to be limited to small prospective and retrospective clinical studies and case reports. There are currently 41 trials published enrolling a total of 162 patients (Table 3). Most patients enrolled have refractory ventricular tachycardia (VT) resulting from non‐ischaemic cardiomyopathy (NICM) or ischaemic cardiomyopathy (ICM). Rarer aetiologies of VT such as cardiac sarcoid (*n* = 4), arrhythmogenic right ventricular cardiomyopathy (*n* = 1), hypertrophic cardiomyopathy (*n* = 2) and cardiac tumours (*n* = 2) have been treated with SBRT.^28^, ^29^, ^30^, ^31^, ^32^ A small number of patients with PVC‐induced cardiomyopathy (*n* = 3) and ventricular fibrillation (*n* = 1) have also been treated with SBRT.^4^, ^33^, ^34^ Of note, SBRT has been used in patients with contraindications to CA, that is mechanical valves (*n* = 9) and LV thrombus (*n* = 5), as well as critically ill patients presenting with electrical storm.^12^, ^35^ Atrial fibrillation has been treated by SBRT in small case series to varying success; however, the main focus of research has been on VA due to the clinical need for alternate more efficacious therapies in this cohort of patients.^36^, ^37^

The workup of patients across clinical studies is not standardised and variable. Most groups utilise invasive electroanatomical maps (EAM) obtained during CA combined with anatomical scar imaging obtained from cardiac CT, cardiac magnetic resonance (CMR) and echocardiography to define VT substrate.^35^, ^38^, ^39^ Some groups have substituted invasive EAMs with dedicated non‐invasive maps, allowing a completely non‐invasive approach.^25^, ^33^ Other groups have employed nuclear medicine scans such as positron emission tomography (PET‐CT) and single photon emission tomography (SPECT) to assist in identifying areas of myocardial viability within scar.^24^, ^40^

The reported planned target volume (PTV) differs markedly across current studies ranging from a median of 51.3 mL to 317.1 mL (Table 3).^25^, ^41^ The large variability in PTV may be explained by several different factors. Firstly, there were differences in anatomical size of VT substrate with initial studies having a higher proportion of NICM than ICM with smaller treatment volumes.^25^, ^35^, ^41^ Secondly, different groups have utilised different strategies in targeting scar. Some studies looked to minimise treatment volumes by targeting SBRT to VT exit sites, whilst others utilised SBRT to target the entire substrate.^4^, ^41^ Thirdly, manual delineation of VT substrate and target volume (TV) contouring likely results in interobserver and intergroup variability.^42^, ^43^ Finally, a range of different margin expansions have been utilised across studies to attain both internal target volume (ITV) and PTV.^38^, ^44^, ^45^

Margin expansions to account for treatment uncertainties vary depending on whether Linac or CyberKnife platforms are employed to deliver SBRT. The CyberKnife platform utilises ICD leads, left ventricular (LV) leads or temporary pacing wires as fiducials to allow marker‐based tracking of respiratory motion (Synchrony, Accuray).^24^, ^38^, ^46^ Cardiac motion has been accounted for in some CyberKnife treatments by incorporating cardiac motion on fluoroscopy and using ECG‐gated CTs.^24^, ^38^ CyberKnife utilises a more conservative 3 mm (range, 0–5 mm) ITV to PTV expansion based on the reported accuracy of CyberKnife for lung/abdomen SBRT.^46^, ^47^ Linac platforms utilise a combined cardiorespiratory ITV obtained from respiratory gated 4D CT. The majority of Linac treatments utilised 5 mm (range, 0–10 mm) expansion of ITV to PTV.^4^, ^25^, ^39^, ^41^

### Efficacy of cardiac stereotactic body radiotherapy

Mirroring the significant differences in workup and treatment of patients in these studies is the variable efficacy of SBRT observed across studies. In contrast with 99.9% reduction in VT burden described by Cuculich in his seminal paper, some groups have reported a reduction in VT burden as low as 31%.^41^, ^44^ Similarly, reduction in ICD therapies has been reported as low as 57% reduction in shocks and 50% reduction in anti‐tachycardia pacing (ATP) therapies.^40^, ^48^

Early suppression of VA within a 6‐week period has been noted across multiple clinical trials. It is apparent that this suppressive effect occurs within a 6‐week period in the absence of fibrosis from post‐mortem results and animal studies.^4^, ^35^, ^39^, ^49^ These data challenge the hypothesis that SBRT achieves its antiarrhythmic effect by solely inducing fibrosis.

Most patients had late recurrence of VA occurring post‐SBRT beyond the 9‐month mark.^35^, ^39^, ^44^, ^49^ EAMs and 12 lead ECGs obtained from these patients following recurrence have indicated viable myocytes within the PTV and also identified new VT circuits in areas adjacent to the PTV.^39^, ^50^ Recurrence within the PTV may reflect resistance of VT substrate to SBRT. Larger PTV size has been correlated with ICM and decreasing efficacy of SBRT for VT, perhaps indicating that 25 Gy is not sufficient to achieve arrhythmia suppression in ischaemic scar.^41^, ^44^ VT circuits adjacent to the PTV may result from scar development from subthreshold dosing of SBRT in viable myocardium.^50^ More consistent and precise clinical target definition with better cardiorespiratory motion management during SBRT delivery may reduce the propensity for late recurrence to occur from VT circuits adjacent to the PTV.

### Potential mechanisms of SBRT

Preclinical studies aimed to utilise radiotherapy to create ablative lesions comparable to CA by destroying cells, inducing fibrosis and producing electrically inert tissue. Fibrosis was dose‐dependent with higher doses utilised (30–55 Gy) to achieve strongly fibrotic, non‐conducting lesions at 3–6 months.^21^, ^22^ A much earlier antiarrhythmic effect has been observed in clinical trials (<6 weeks) utilising a lower 25 Gy dose. The therapeutic effect of radiotherapy therefore cannot be explained by fibrosis alone.

Immediate blockade of conduction in the AV node has been achieved in large animal study with very large doses of SBRT (160 Gy).^51^ This functional blockade of conduction may be achieved through disruption of cardiomyocyte electrical conduction in the absence of cell death. Given functional conduction blockade can occur acutely and in the absence of fibrosis, it is plausible that this may contribute to the early antiarrhythmic effect noted in clinical trials.^51^ Further investigation will need to be done to determine the minimum dose required to achieve this effect in ventricular tissue and the underlying mechanisms.

Lower doses of radiation (10–25 Gy) in animal infarct models have been associated with improved conductivity within scar tissue reducing vulnerability for re‐entrant arrhythmias such as VT. Animal studies have shown that improved conduction may be mediated by increased connexin 43, a major ventricular gap junction subunit, and voltage‐gated sodium channels (Na_V_1.5).^52^, ^53^, ^54^, ^55^ Connexin 43 upregulation in these studies was notably associated with a decrease in VT inducibility.^53^, ^54^ The changes above have been observed as earlier as 2 weeks post‐treatment following radiotherapy accounting for the early antiarrhythmic effect observed in current clinical trials.^52^, ^55^

Reversal of cardiac remodelling and increasing ejection fraction is associated with reduced risk of VA and mortality.^56^ CMR data in a subset of patients 3 days (*n* = 9) pre (EF 18.6% ± 3.9%)‐ and post‐treatment (EF 32.3% ± 4.8%) demonstrated an early but persisting improvement in ejection fraction at 3 months (32.2% ± 7.3%).^26^ Retrospective analysis of the ENCORE‐cohort (*n* = 42) revealed that ≥40% of the heart outside of the PTV received ≥5 Gy. The mean heart dose extrinsic to the non‐targeted areas of myocardium was 5 Gy.^57^ The effect of 5 Gy cardiac irradiation was investigated in 3 murine heart failure models by Pedersen *et al*., who found an improvement in ejection fraction and end diastolic volume in the radiotherapy group compared to sham.^57^ Although VT suppression may account for the improvement in ejection fraction observed in ENCORE VT, these data raise the question whether low‐dose radiotherapy also plays a role in reversing cardiac remodelling thereby reducing the risk of VT.

### Known early and late toxicities from cardiac stereotactic body radiotherapy

Historically, our knowledge of cardiac toxicity from radiotherapy stems from clinical experience with fractionated treatment in lymphoma and breast cancer.^58^ Acute cardiac inflammation from radiotherapy manifests in myocarditis or pericarditis. Most radiation‐induced cardiotoxicity appears to be delayed, usually developing 10–40 years after treatment.^58^ The dose of radiation, volume of heart and extent of involvement of coronary arteries are risk factors associated with development of cardiac toxicity. Common toxicities include early onset coronary artery disease, valvular heart disease, complete heart block, heart failure from diastolic dysfunction and pericardial fibrosis.^58^

Adverse effects due to cardiac SBRT are summarised in Table 1. Transient early adverse effects such as fatigue, dizziness, nausea and hypotension are common, occurring in up to 23% of patients, and usually resolve spontaneously (grade 1). There has been 1 case of ICD reset to factory settings post‐SBRT which has been reported^39^ (grade 2). Rare early delayed adverse effects which have been reported post‐SBRT are fatal heart failure exacerbations and refractory electrical storm (grade 4 and 5).^35^, ^45^, ^59^ These adverse effects may reflect the natural history of disease in this patient cohort, but a SBRT‐related adverse effect is unable to be excluded.

**Table 1:** Adverse effects from SBRT

Adverse events	Manifestation	Clinical grade (CTCAE)	Management
Transient early adverse events	Fatigue, dizziness, nausea and hypotension	1	Spontaneous resolution
Late delayed toxicities	Pneumonitis	2	Steroid therapy
	Pericarditis Pericardial effusions	3–4	Medical therapy and rarely require pericardiocentesis
	Mitral valve regurgitation	4	Valve surgery
	Aortic regurgitation	4	Valve surgery
	Left thoracic chest discomfort	3	Steroids and cervical nerve root block
	Gastroesophageal fistula	4	Surgical correction
	Oesophageal‐pericardial fistula	5	Gastrostomy/mortality from fatal bleeding

Common late delayed toxicities include radiation‐induced pneumonitis and pericarditis (grade 2 and 3).^33^, ^35^, ^39^, ^44^, ^48^, ^59^ Pneumonitis occurs in 11–29% of patients and generally resolves with steroid therapy. Pericarditis and associated pericardial effusions occur in 17–29% of patients and usually respond to medical therapy and rarely require pericardiocentesis. There have been two cases of severe mitral valve regurgitation which required surgery (grade 4) and one case of moderate aortic regurgitation (grade 3) which were likely radiation‐mediated.^28^, ^60^ Progress CT scans and echocardiograms post‐SBRT are important to ensure that these complications are detected, monitored and treated. There has been 1 case of left thoracic chest discomfort reported which improved with steroids and cervical nerve root block (grade 3).^39^

Rare late delayed toxicities from SBRT are gastroesophageal fistula (grade 4) and oesophagopericardial fistula (grade 5).^26^, ^61^ The oesophagopericardial fistula patient presented with oesophagitis that responded to antacids 18 days post‐SBRT. At 6 months, the patient presented with dysphagia and a large ulcer was diagnosed in the terminal part of the oesophagus on endoscopy. Endoscopic gastrotomy was performed as surgery was deemed to high risk. The patient had a fatal bleeding event 3 months post‐gastrostomy. The oesophageal dose limits were not exceeded in this case. The gastropericardial fistula occurred 2.4 years post‐SBRT. It was corrected with surgical treatment. Patients receiving radiation therapy to inferior or inferolateral wall, which lies near the gastric tract, must therefore be monitored carefully for this complication.

## Current workflow

### Patient selection

Currently, SBRT remains an experimental therapy that is offered as part of clinical trials or compassionate access to patients that have VA that is refractory to antiarrhythmic drug escalation and who have failed or are not candidates for catheter ablation. As shown in Table 3, which summarises the data to date supporting use of SBRT, it can be an effective treatment modality in such cases, and its non‐invasive nature makes it particularly suited to treat highly comorbid patients who cannot tolerate general anaesthesia or hospitalisation or have inaccessible scar. The outcome data as demonstrated above remain limited to prospective case studies with no randomised control data to support its use.

### Determining clinical target

**Fig. 2:**
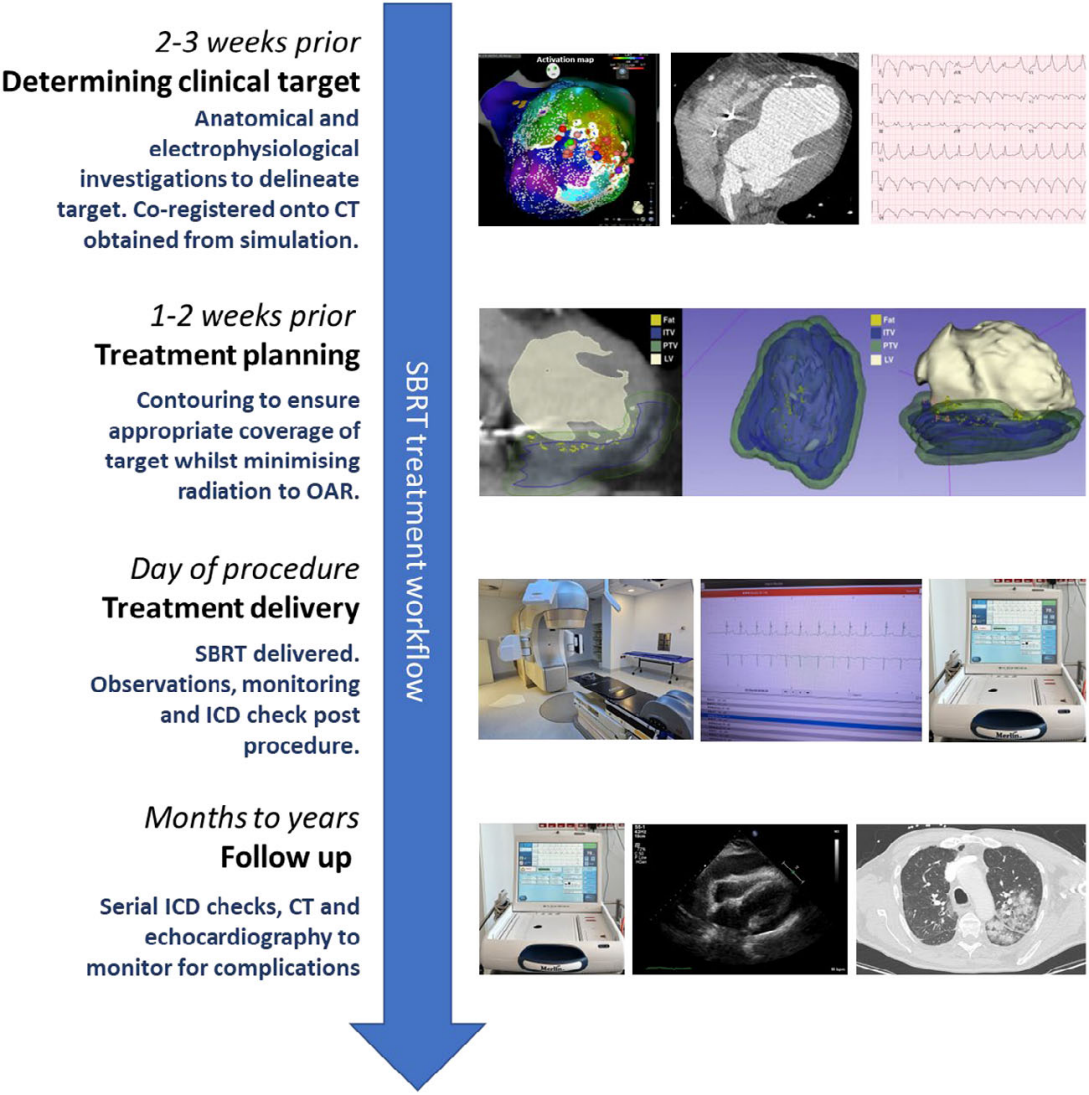
SBRT treatment workflow. Reprinted [adapted] from Qian *et al*.^129^

A combination of anatomical and electrophysiological investigations allows delineation of arrhythmic substrate driving VA. In patients who have failed catheter ablation, EAM information can be integrated with cardiac imaging to provide understanding of arrhythmogenic substrate^62^, ^63^ (Fig. 2). Electrocardiographic imaging (ECGi) or CardioInsight (Medtronic, Minnesota, United States) utilises an electrode vest, with 60–252 electrodes, in combination with CT scan, can non‐invasively map the VA exit in centres where this is available.^64^

Imaging techniques available for localisation of scar in structural heart disease include CMR, cardiac CT and radionuclide imaging. CMR provides excellent soft tissue characterisation, visualising myocardial scar with great precision.^65^ The utility of CMR can be limited due to artefacts created by ICDs leads; however, wide‐band filters now mitigate this to some extent.^66^ Regions of myocardial thinning and putative corridors for VA circuits can be extracted using image processing software (ADAS 3D, Galgo Medical, Barcelona, Spain or In heart, Bordeaux, France) and can be imported into radiotherapy planning software.^67^, ^68^ Cardiac CT provides higher spatial resolution allowing excellent characterisation of cardiac anatomy.^69^, ^70^ It can identify areas of significant wall thinning, fibrosis, fat and calcium which are associated with VA substrate.^71^, ^72^ The anatomical information obtained from cardiac CT can be combined with delayed contrast administration.^70^ Radionuclide imaging, such as SPECT and fluorodeoxyglucose‐positron emission tomography (FDG‐PET), can detect areas of scar based on metabolic activity. Areas of rapid transition from low to normal metabolism on FDG‐PET, indicate border zones between normal and scar tissue, have been correlated to VT exit sites on EAMs.^73^

### Radiotherapy simulation, target volume creation and treatment planning

Patients undergo a treatment simulation session in the radiation oncology CT suite where they are immobilised in the position in which they will receive radiation. Vac‐bags, abdominal compressors and overhead arm extensions are used to assist in immobilisation in a reproducible position. A 4D CT is performed and the average intensity projection typically serves as the anatomic reference for treatment planning to account for respiratory cycle motion in free breathing treatments.^74^

Electrophysiological and anatomical information is co‐registered with the simulation CT to define a TV (Fig. 2). This is contoured by a collaborative effort between the electrophysiologist and radiation oncologist utilising the anatomic (MRI, CT, radionuclide) and electrophysiological information (ECG, EAM and ECGi).^42^, ^75^ An ITV is generated from the TV which accounts for maximum range of cardiorespiratory motion demonstrated from the 4D‐CT. PTV is generated as a small volumetric expansion (≤5 mm) from the ITV which accounts for geometric uncertainties.^42^ The contouring process is time‐consuming as it often requires manual comparison between imaging studies and mapping data as well as consideration of adjacent radiosensitive organs.^42^ There is continued development in tools to automate integration of electrophysiological maps with radiation planning software; however, these tools require further clinical validation (Table 2).^76^

**Table 2:** Comparison of cardiac fusion papers

Cardiac fusion papers	Software translation	EAM software	Plug in	File conversion	Significance
Brett *et al*. (2020)^77^	EAM to radiation planning software	CARTO 3	SlicerRT	Mesh→VTK→DICOM	Robust performance when compared to ADAS VT
Hohman *et al*. (2020)^78^	EAM to radiation planning software	CARTO 3, EnSite, Rhythmia	EAMapReader (3D slicer)	Mesh/XML→DICOM	Compatible with multiple EAM systems
Mayinger *et al*. (2023)^79^	EAM to radiation planning software	CARTO 3, Rhythmia, EnSite	CARDIO‐RT	Mesh→DICOM	Compatible with multiple EAM systems
Santos‐Ortega *et al*. (2022)^80^	EAM to radiation planning software	EnSite	ADAS‐3D	N.R.	MRI fused with EAM and radiotherapy planning CT
Qian *et al*. (2022)^41^	Cardiac CT segmentation to radiation planning software	N/A	MUSIC (3D slicer)	N.R.→DICOM	4D CT segmented into 3D volumes
Peichel *et al*. (2021)^81^	EAM to radiation planning software	CARTO 3	In house tool (3D slicer)	Mesh→VAK→DICOM	
Lee *et al*. (2021)^45^	EAM to radiation planning software	Unspecified EAM software	Mimics innovation suite	Mesh→VAK→DICOM	
Van der Ree *et al*. (2022)^82^	Cardiac CT segmentation to radiation planning software	N/A	In house tool	N.R.	
Hohman *et al*. (2023)^83^	EAM to radiation planning software	CARTO 3, EnSite Velocity/Precision, Rhythmia HDx	EAMapReader (3D slicer) Cardio‐RT	Mesh/XML→DICOM	Good agreement between Cardio‐RT and ADAS‐3D
Wang *et al*. (2023)^76^	EAM to radiation planning software	CARTO 3, Rhythmia HDx, ECGi	HeaRTmap (3D slicer)	Mesh/XML→DICOM	ECGi compatible
Oh *et al*. (2023)^84^	EAM to radiation planning software	CARTO 3	In house tool	Mesh→DICOM	
Rigal *et al*. (2023)^85^	EAM/CT‐PET to radiation planning software	CARTO 3	In house tool	Mesh→VTK→DICOM	Integration of cardiac CT‐PET with EAM to delineate target

**Table 3:** Clinical studies of SBRT for VA to date

Reference	Patient characteristics (sample size, gender, age)	Dose, procedure length	Delivery system	PTV size (median, range)	ITV to PTV expansion	Follow‐up	Recurrence	SBRT complications	Mechanistic findings
Cvek *et al*. (2014)^112^	*N* = 1 female; Age 72	25 Gy in 1 fraction; 114 min	CyberKnife	N.R.	N.R.	4 months	No recurrence at 4 months	None	Minimal elevation of troponin T serum levels detected 10 days after treatment
Loo *et al*. (2015)^24^	*N* = 1 male; Age 71	25 Gy in 1 fraction; 90 min	CyberKnife	N.R.	N.R.	9 months, 2 month blanking period	Reduction in VT burden from 562 to 52 episodes per month at 3 months	None Patient died 9 months later due to exacerbation of COPD and recurrent VT	Cycle length of VT decreased from 380–411 ms to 470 ms post‐SBRT
Cuculich *et al*. (2017)^25^	*N* = 5 (4 male, 1 female); Age 66 (60–83)	25 Gy in 1 fraction; 15 min	Varian TrueBeam	51.3 mL, 17.3–81 mL	5 mm expansion	12 months, 6‐week blanking period	99% reduction in total VT episodes seen in ¾ evaluable patients Recurrence in ¼ evaluable patients	1 patient died of a stroke 3‐weeks after SBRT. Remains unclear if this was related to SBRT	No CT evidence of changes to myocardium or coronary arteries 12 months post‐treatment in patients Post‐mortem pathological assessment of the deceased patient revealed viable myocardium with ectatic blood vessels on the interface of dense scar – often explained by the acute vascular injury. However, no signs of acute vasculitis, tissue oedema, acute myocyte necrosis, haemorrhage or acute inflammation
Jumeau *et al*. (2018)^113^	*N* = 1 male; Age 75	25 Gy in 1 fraction; 45 min	CyberKnife	21 mL	N.R.	4 months	No recurrence at 4 months	None	N/A
Haskova *et al*. (2019)^31^	*N* = 1 male; Age 34	25 Gy in 1 fraction; N.R.	CyberKnife	N.R.	N.R.	8 months	Gradual reduction in VT episodes. No recurrence at 8 months	None	N/A
Zeng *et al*. (2019)^32^	*N* = 1 male; Age 29	24 Gy in 3 fractions; N.R.	CyberKnife	71.2 mL	N.R.	4 months	Complete reduction from 189 to 0 VT episodes/day after 2 months No recurrence at 4 months	None	N/A
Scholz *et al*. (2019)^34^	*N* = 1 male; Age 53	24 Gy in 1 fraction; 5 min	Elekta Versa	55.8 mL	2 mm expansion	2 months	No VT episodes at 2 weeks post‐SBRT No recurrence at 2 months	None	N/A
Neuwirth *et al*. (2019)^38^	*N* = 10 (9 male, 1 female); Mean age 66 (61–78)	25 Gy in 1 fraction; 68 min (45–80 min range)	CyberKnife	22.1 mL, 14.2–29.6 mL	0 mm expansion	28 months, 90‐day blanking period	An average of 87.9% reduction in VT episodes from 70.7 to 8.7 episodes/month. 2 patients with no response; Recurrence in 8/10 patients	4 patients with nausea associated with acute toxicity 1 patient with possible grade 3 late toxicity (mitral regurgitation at 17 months) 3 unrelated deaths	No signs of significant elevation of troponin during follow‐up
Krug *et al*. (2019)^114^	*N* = 1 male; Age 78	25 Gy in 1 fraction; 15 min	Varian TrueBeam	42.2 mL	5 mm expansion	2 months	Reduction from 20 to 6.4 VT episodes/month; Recurrence at 2 months	Periprocedural nausea with a single episode of vomiting; Patient died 57 days post‐treatment due to sepsis associated cardiac circulatory failure.	Clinical autopsy revealed diffuse fibrosis throughout heart (no evident differences between target tissue and the control: posterior cardiac wall tissue). Specific to the target area, there was a 2 mm region of fresh necrosis and no evidence of a demarcating inflammatory reaction
Robinson *et al*. (2019)^4^	*N* = 17 (15 males, 2 females); Median age 66 (49–81)	25 Gy in 1 fraction; 15.3 min (5.4–32.3 min range)	Varian TrueBeam (*n* = 3) and Varian Edge (*n* = 16)	98.99 mL, 60–298.8 mL	5 mm expansion	24 months, 6‐week blanking period	97% reduction from median of 119 to 3 VT episodes/month Recurrence in 11/16 evaluable patients at 6 months	1 patient with pneumonitis 2 patients with delayed pericarditis/ Effusion 1 patient died from unrelated accident 17 days post‐SBRT	N/A
Lloyd *et al*. (2019)^48^	*N* = 10 (7 males, 3 females), Median age 61 (51–78)	25 Gy in 1 fraction; 30 min appointment	Varian Edge	81.4 mL, 45–238 mL	1–5 mm expansion	6 months	A mean reduction of 94% in total seconds of detected VT in 9/9 evaluable patients. One patient with no response to SBRT. Most of patients responding in first 2 weeks No recurrence at 6 months	2 patients with mild pneumonitis. 1 patient requiring resuscitation with slow VT (below treatment zone of her device) during SBRT treatment; 1 patient required heart transplant after no response to SBRT	Observed disruption of gap junctions on electron microscopy specimens (possibly explaining the relatively acute treatment response)
Qian *et al*. (2019)^36^	*N* = 2 (1 male and 1 female), Age 59 and 55	25 Gy in 1 fraction; 90 min for each treatment	CyberKnife	48.87 mL and 54.5 mL	N.R.	6 months	Recurrence of atrial fibrillation at 6 months in 1st patient. No recurrence in 2nd patient at 6 months	Nil complications	
Park *et al* (2020)^29^	*N* = 1; Male; Age 76	24 Gy three fractions; N.R.	Varian Clinac iX	N.R.	10 mm expansion	6 months	2 recurrences of VT resolving with ATP. No further ICD shocks	Mild pulmonary fibrosis on CXR	N/A
Qian *et al*. (2020)^50^	*N* = 1 male; Age 77	25 Gy in 1 fraction; N.R.	N.R.	N.R.	N/A	4 months	Reduction from 12 to 4 cardioverter‐defibrillator shocks in 3 months. No recurrence at 4 months	Tapering of antiarrhythmic medications after 4 months revealed new recurrent VT at treatment border zone	N/A
Shoji *et al*. (2020)^37^	*N* = 3 (2 male, 1 female), Age 76, 67 and 81	25–35 Gy in 1 fraction, N.R.	CyberKnife	78.5 mL, 51.5–111 mL	3 mm expansion	24 months	AF in patients with metastatic cancer. Recurrence of atrial fibrillation occurred in remaining 2 patients who survived until 24 months	1 unrelated patient death 4 days after procedure from cancer progression	
Gianni *et al*. (2020),^44^	*N* = 5 males, Mean Age 63 (45–76)	25 Gy in 1 fraction; 82 min (45–80 min range)	CyberKnife	173 mL, 96–184 mL	3 mm expansion	12 months	Reduction in 4/5 patients from 6.4 to 4.9 VT episodes /month; Recurrence in all patients 6 months later	None; 2 patients died of advanced heart failure	N/A
Mayinger *et al*. (2020)^115^	*N* = 1 male; Age 71	25 Gy in 1 fraction; 24 min	MR Linac	73.6 mL	2 mm	3 months	100% reduction in episodes at 1 week; No recurrence at 3 months	Aggravation of VT and prolonged ES 24 h post‐SBRT, ceasing after 48 h following high dose of dexamethasone	N/A
Yugo *et al*. (2020)^40^	*N* = 3 (2 males, 1 female), Age 72 (68–83)	25 Gy in 1 fraction; N.R.	Varian TrueBeam	92.95 mL. 63.9–106.6 mL	2 mm	13 months, 6 week blanking period	Mean reduction from 140 to 54 VT episodes in 2/3 patients Recurrence in 3/3 patients at 6 weeks	None; 3/3 unrelated patient deaths due to comorbidities	N/A
Peichl *et al*. (2020)^81^	*N* = 1; Male; Age 66	2 doses of 25 Gy; N.R.	CyberKnife.	21 and 18 mL respectively for each case	3 mm	12 months	1st SBRT guided by electroanatomical mapping and CT failed to suppress ventricular tachycardia. Subsequent electroanatomical mapping showed evidence of scar adjacent to area of earliest activation of VT. 2nd SBRT delivered with new integration software. Smaller target volume allowed addition of 3 mm margin to PTV increasing the probability of achieving transmural lesion. No further VT at follow‐up at 12 months following 2nd SBRT	None	Nil
Chin *et al*. (2021)^116^	*N* = 8 males; Mean age 70 (65–86)	25 Gy in 1 fraction; 18.2 ± 6 min	Brainlab Novalis Tx	84.9 mL, 21.1–190.7 mL	6–8 mm margin	8 months	Mean reduction from 17 to 4.1 episodes/month. Resolution in 3/6 evaluable patients by 3 months; Recurrence in 4/6 evaluable patients at 8 months	No acute complications 3 unrelated patient deaths	N/A
Dusi *et al*. (2021)^117^	*N* = 1 male; Age 73	25 Gy in 1 fraction; N.R.	N.R.	27.7 mL	N.R.	2 months	Reduction from 121 episodes/month to 5.5 episodes/month	No acute complications. Patient passed away from septic shock 2 months post‐SBRT	N/A
Carbucicchio *et al*. (2021)^33^	*N* = 7 males; Mean Age 70 (63–77)	25 Gy in 1 Fraction; 31 ± 6 min	Varian trilogy	198.3 mL, 138–225 mL	N.R.	6 months	4/4 evaluable patients showed significant decrease from 29 to 2 VT episodes/month at 6 months	1 episode of pulmonary fibrosis. 1 episode of nausea and vomiting 3 unrelated patient deaths	N/A
Lee *et al*. (2021)^118^	*N* = 1; Male; Age 11	25 Gy single fraction; N.R.	Elekta Versa HD	N.R	5 mm	3 months	No VT at 3 months after SBRT and bilateral stellate ganglion blockade	None	N/A
Donnelly *et al*. (2021)^119^	*N* = 1; Male; 51 M	25 Gy single fraction; N.R.	N.R.	N.R.	5 mm	Not documented	Decrease in PVC burden from 21% to 5% on follow‐up with improvement of LV function to 45%–50% (decreased due to PVC induced cardiomyopathy)	None	
Ho *et al*. (2021)^30^	*N* = 7 (6 males, 1 female); Age 55 (23–80)	25 Gy in 1 Fraction; N.R.	Varian TrueBeam	52.1 mL, 14.4–92.6 mL	5 mm	14.5 months	Significant reduction in VT episodes Recurrence in 4/6 evaluable patients	No acute complications; Left ventricular apical thrombus prevented one patient from receiving SBRT. One patient with grade 1 pericardial effusion 6 months post‐treatment One unrelated death due to hepatic failure	N/A
Gerard *et al*. (2021)^120^	*N* = 2; Male; Age Both 65	25 Gy single fraction; 20 and 24 min respectively	Varian TrueBeam	103 and 66.4 mL respectively for each case	N.R.	17 and 12 months	Case 1 had ES in context of pneumonia 10 months after treatment. At 17 months one recorded VT event and no ICD shocks. Case 2 had no further VT at 12 month follow‐up	Case 1 had mild oesophagitis treated with antacids	N/A
Aras *et al*. (2021)^12^	*N* = 1; Male; Age 58	25 Gy single fraction; 7.5 min	Varian TrueBeam.	N.R.	5 mm	10 months	Significant reduction in VT episodes following SBRT	No complications.	N/A
Lee *et al*. (2021)^45^	*N* = 7 (4 males, 3 females); N.R. (60–70s)	25 Gy in 1 Fraction; 33 min (30–60 min)	Varian medical systems and Elekta	89.5 mL, 57.5–139 mL	3–5 mm margin	6 months	85% reduction in VT episodes at 6 months post‐SBRT in 5/5 observable patients Short term recurrence observed	None; Three patient deaths from progressive heart failure, two dying within 4 weeks post‐treatment	Autopsy obtained from patient that died within 4 weeks of treatment demonstrating increased vascularity in viable myocardium and previous fibrosis due to myocardial ischaemia No other histologic features of acute necrosis, thrombosis, changes to blood vessel wall, fibroblast proliferation or nuclear atypia to suggest radiation exposure were evident
Qian *et al*. (2022)^41^	*N* = 6 Males; Median age 72 (70–83)	25 Gy in 1 Fraction; 13.8 min (11.0–15.0 min)	Varian TrueBeam	317.1 mL, 262–345.1 mL	5 mm margin	18 months	Insignificant reduction from 42 (IQR 19–269) to 29 (IQR: 0–81) VT episodes in 6 months Significant reduction in device shocks from 12 (IQR: 3–9) to 0 (IQR 0–1) Recurrence in 4/6 patients at 213 (IQR 105–306) days post‐treatment	3/6 patients with possible adverse effects including pneumonia, heart failure exacerbation, asymptomatic pericardial effusion; 3 patient deaths due to end‐stage heart failure	Substrate modification approach targeting regions of wall thinning, myocardial fat and calcification in ischaemic cardiomyopathy utilised
Haskova *et al*. (2022)^121^	*N* = 3; Male; Age 66 (34–77)	Single dose of 25 Gy; N.R.	CyberKnife	Redo PTVs –21.2, 23.4 and 43.4 mL respectively for each case	3 mm for case 2. N.R. for case 1 and 3	22–32 months	Patient 1 and 2 underwent redo SBRT at 20 and 24 months post‐index SBRT. No further VT at 22 and 32 months following 2nd SBRT Patient 3 had SBRT for recurrent VT 4 months after index SBRT. Required additional CA for slow VT to render non‐inducible. At 2 month mark died of progressive heart failure with no further VT	None	N/A
Wight *et al*. (2022)^59^	*N* = 14 (10 Males, 4 Females); Mean Age 61 (50–78)	25 Gy in 1 fraction; N.R.	Varian TrueBeam	N.R.	1–5 mm expansion	7 months	Patients had a 59% reduction in VT, 39% reduction in ATP and a 60% reduction in shocks	Pneumonitis in four of the 14 patients Two patients died shortly after SBRT, one died post‐heart transplant and another had ES and haemodynamic instability leading to death	N/A
Miszczyk *et al*. (2022)^122^	*N* = 1 Male; 51 year old	25 Gy in 1 fraction; 33 min	Varian Edge	88.7 mL	3 mm expansion	12 months	No recurrence	Antiarrhythmic response that lasted almost a year, until a heart failure exacerbation necessitated a heart transplant	No evidence of fibrosis Despite a complete treatment response, there was no homogenous transmural fibrosis in the irradiated region, and the overall presentation of the heart was similar to other transplanted hearts of patients with advanced heart failure
Ninni *et al*. (2022)^35^	*N* = 17; Males 13; Females 4; Mean age = 67 ± 12.8	25 Gy in 1 fraction; 30 min	CyberKnife	53.3 mL, 20.0 mL –186 mL	5 mm expansion	Median 12.5 (10.5–17.8) months	High rate of patients presenting late VT recurrence after SBRT. 40% of these patients presented VT recurrences related to single VT episodes, and 30% of patients presented VT recurrences associated with ICD shocks at 18 months	Two patients died during the blanking period (<6 weeks). 1 patient died of cardiogenic shock and the other with refractory ES.2 patients died beyond 6 weeks. 1 patient died from AKI, metabolic acidosis and electromechanical dissociation. The other died from refractory cardiogenic shock	Late VT recurrences were unrelated to ES and were associated with increase in cycle length in VT in two‐thirds of the patients
Bernstein *et al*. (2022)^123^	*N* = 1; Male; Age 75	25 Gy in single fraction; N.R.	N.R.	87.9 mL	5 mm	6 months	No VT recurrence	N/A	N/A
Cozzi *et al*. (2022)^124^	*N* = 1; Male; Age 81	25 Gy in single fraction; 15 min	Varian True Beam	122.5 mL	5 mm	7 months	No VT recurrence	None	N/A
Huang *et al*. (2022)^125^	*N* = 1; Male; Age 63	12 Gy in single fraction; 24 min	Versa HDTM	65.75 mL	5 mm	15 months	At 6 months, recurrence of VT from different anatomically area requiring ATP and ICD shock. VT was mapped and ablated. Following placement of CRT device and ablation marked decrease in VT burden	N/A	N/A
Vaskovskii *et al*. (2022)^126^	*N* = 1; Male; Age 57	Single dose of 25 Gy; N.R.	Varian TrueBeam	46 mL	5 mm	6 months	No further VT episodes after 2 month mark post‐procedure	Nil	N/A
Aras *et al*. (2023)^49^	*N* = 8, 8 Males; Mean age 58 ± 14	25 Gy in 1 Fraction; 5.6 min (3.6–7.45 min)	Varian TrueBeam	157.44 mL, 70.5–272.7 mL	5 mm	8 months	All patients demonstrated VT recurrences; however, ICD and anti‐tachycardia pacing therapies were significantly reduced with SBRT. The 2 weeks to 3 months period outcomes were favourable. After 6 months, one patient was ICD therapy‐free and the remaining patients demonstrated VT episodes	2 pericardial effusions. 1 asymptomatic and 1 resolved with medical management Four patients died during follow‐up. 1 patient had end stage heart failure and recurrent VT. 3 deaths due to unrelated causes	N/A
Van Der Ree *et al*. (2023)^39^	*N* = 6; 6 Males; Age 73 (54–83)	Single dose 25 Gy; 4.6 min (3.6–5.2 min)	Agility linear accelerator (Elekta)	187 mL, 93–372 mL	5 mm	12 months	Met primary outcome of ≥50% reduction in VT in 4 patients Median number of treated VT episodes reduced from 31 (range 8–138) before treatment to 9 (range 0–109) after treatment Recurrence occurred in 5 (83%) of patients at 12 months	1 patient had radiation induced electrical reset of ICD 1 patient developed severe thoracic chest wall within the field requiring steroids and cervical nerve block 2 patients developed pericardial effusions responding to conservative management 1 patient had pneumonitis 1 patient developed intracardiac thrombus which was possibly radiation related	N/A
Scanavacca *et al*. (2023)^127^	*N* = 1; Male, Age 53	Single dose of 25 Gy; 15 min	N.R.	74.5 mL	N.R.	12 months	No recurrence in 12 months	None	N/A
Van Der Ree *et al*. (2023)^28^	*N* = 1, Female, Age 47	Single dose 20 Gy to proarrhythmic substrate; CyberKnife. 2 x 2 Gy low‐dose whole heart irradiation	Tomotherapy HAD treatment system	PTV 16 mL 1162 mL for whole heart immunomodulatory radiotherapy.	3 mm	55 months	Reduction in sustained VT episodes by 95% and NSVT episodes by 58%. At 7 months presented in ES due to influenza. Following seventh CA only 2 sustained episodes requiring ATP in following 38 months PET CT demonstrated regression of hypermetabolic activity with no active signs of cardiac sarcoidosis at 55 months	Moderate aortic regurgitation	Reduction in inflammatory disease activity on PET‐CT

Treatment planning is then performed following standard SBRT principles. Dose is highly conformal with prescribed dose covering an isodose allowing for steep gradients and a high maximum inside ITV. Both intra‐ and extracardiac organs‐at‐risk (OAR) dose tolerances adhere to known constraints.^86^ Example beam arrangements and dose‐volume histograms for conventional linacs were reported by Knutson *et al*.^86^ Technical details of reported radiotherapy treatments have been summarised by Lydiard *et al*.^87^

### Accounting for cardiorespiratory motion

Accounting for cardiac motion during the delivery of SBRT remains a challenge.^42^, ^88^ Reducing or tracking cardiac motion during SBRT will allow for decreased PTV and reduced collateral damage to structures at risk. Recent studies looking at cardiac motion during systole and diastole during 4D CT estimate average displacements of 2–3 mm with maximum displacements of 5–6 mm during breath holding conditions.^89^, ^90^, ^91^ During free breathing treatment, the target motion envelope is typically dominated by respiratory rather than cardiac motion.^92^, ^93^ ITV expansions like those used in thoracic SBRT then encapsulate motion sufficiently. Organs at risk (OAR) such as stomach may require a planning risk volume (PRV) to ensure they are kept within dose tolerances during treatment delivery. If PRV cannot be planned to tolerance, additional motion management strategies may be required (breath hold, beam gating, etc.) or re‐simulation after fasting.^94^

Abdominal compression is used commonly in thoracic SBRT to force shallow breathing, thus reducing tumour motion in thoracic and abdominal tumour sites. Mannerberg *et al*. assessed the effect of abdominal compression on cardiac movement in lung cancer patients undergoing SBRT.^95^ Most patients had a large (≥3 mm) motion reduction in LV wall movement in the superior/inferior axis. However, a small number of patients had increased heart motion. Further to this, upward movement of the stomach towards the inferior wall may lead to an increased risk of gastric complications.

Feasibility studies on gel phantoms have demonstrated that it is technically feasible to safely deliver effective radiotherapy gated to cardiac cycle using ECG signals.^96^, ^97^ Image acquisition in CT, CMR and echocardiography is often gated to the ECG.^98^, ^99^, ^100^ CT and CMR images are often acquired in mid diastole where cardiac motion is minimal to reduce the amount of motion artefact.^101^ Integration of ECG‐gated CMR, echocardiography and CT images during the delivery of ECG‐gated SBRT may improve target definition and tracking accuracy, allowing for further reduction in ITV margins.^102^, ^103^ Validation of these technologies in larger clinical trials will be required.

### Treatment delivery

Patients attend radiotherapy treatment as outpatients. They are positioned and immobilised on the linear accelerator (Linac) couch to replicate the simulation session. Alignment of the patient can be verified by fluoroscopic imaging and cone beam CT with fine adjustments made. Treatment is delivered in conscious patients who then undergo post‐therapy observations and monitoring prior to discharge (Fig. 2).

Conventional Linac‐based systems have the advantage of being readily accessible and allowing rapid delivery of therapy in 5–6 min.^104^, ^105^ For highly comorbid patients with concomitant heart failure who are unable to lie flat, patient tolerability is a crucial factor, especially since movement during SBRT procedures can affect the efficacy and safety of the treatment. In a recent head‐to‐head retrospective comparison, Linac‐based systems showed superiority in sparing distal critical structures compared to CyberKnife due to lower treatment times.^105^ CyberKnife systems have limited availability, but have the advantage of tracking patient movement in real time utilising internal fiducials (such as an RV defibrillator lead) to make minute adjustments to the accelerometer that delivers radiotherapy. CyberKnife can access multiple oblique angles allowing greater flexibility in target volume conformations. Hence, CyberKnife has better dose coverage and dose homogeneity with steeper dose gradient which may give it the edge over conventional Linac‐based systems when targeting basal structures in close proximity to critical structures.

### ICD considerations

Ionising radiation can oxidise insulators within semiconductors that are housed in contemporary ICDs. Newer devices are therefore more susceptible to the effects of radiation which results in a malfunction rate of 3%–7%.^106^, ^107^, ^108^ Malfunctions mainly consist of resets of the device which can be avoided by minimising neutron producing radiation (≤10 mV).^106^, ^107^, ^108^ High dose rates and cumulative dose can also cause transient or permanent damage and these should be kept within manufacturer and best practice guidelines.^109^ High dose rate flattening filter free (FFF) beams are commonly used for cardiac SBRT, and care should be taken to avoid primary beam passing through the ICD and consider using <10MV beam energy as a conservative approach.^110^ Patients presenting for SBRT should have device interrogation prior to undergoing radiotherapy to determine baseline settings of the device and lead parameters. They should be monitored on telemetry during the delivery of SBRT to monitor for any signs of device malfunction. They should undergo a repeat device interrogation following SBRT. One important consideration is that VA recurrence post‐SBRT is often at a slower cycle length.^33^, ^35^, ^39^, ^42^, ^111^ Correspondingly, monitor and therapy zones on ICDs must be adjusted down to allow detection and appropriate treatment.

### Follow‐up

Regular surveillance follow‐up with electrophysiologists and radiation oncologists is necessary post‐SBRT treatment (Fig. 2). From an electrophysiology perspective, patients require an ICD check to assess for arrhythmia response with appropriate adjustment of antiarrhythmics and therapy zones. Transthoracic echocardiography will be required to monitor LV function and for pericardial effusions. Up titration of medications and optimisation of heart failure is also paramount to this cohort of patients. From a radiation oncology perspective, monitoring for the development of pneumonitis both clinically and with CT scans where appropriate.

## Areas for future research

**Fig. 3:**
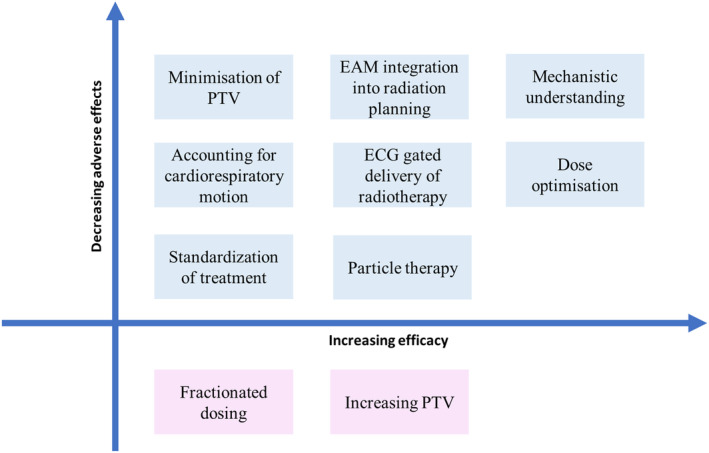
Future directions for the development of SBRT balancing the risks of adverse effects and efficacy.

Originally, SBRT was thought to mediate conduction block through radiation‐induced fibrosis; however, current clinical data question this hypothesis. Mechanistic understanding will be important in determining the optimal dose to achieve VA suppression and exactly what volume of scar needs to be targeted. Standardisation of treatment from patient selection, workup and radiation planning to delivery of radiotherapy will be necessary to further develop this novel treatment modality. Development of automated tools that allow integration of EAM, anatomic and functional imaging during radiation planning will play an important role in ensuring quality assurance through the generation of precise and reproducible TVs. Improved tracking of cardiac motion and ECG‐gated radiotherapy will minimise inaccuracies and ensure critical structures are incorporated into the PTV during radiation delivery. Biomarker verification and development may establish endpoints, other than arrhythmia recurrence, that are predictive of biological and electrophysiological response in scar and a long‐term therapeutic effect. Longer term follow‐up will be important to evaluate for toxicity and impact on cardiac function (Fig. 3).

SBRT must therefore be tested in larger randomised clinical trials with longer follow‐up to better understand the risks and benefits associated with this novel approach. In patients presenting with electrical storm, management and resolution of the cascade of recurrent ICD shocks, sympathetic tone and worsening dyssynchrony that drives further VT may have partially accounted for the reduction in VT observed in these single arm studies, rather than the efficacy of SBRT. Randomisation in comparison with standard of care (CA) will be critical to adjust for this and other important confounders to give a true measure of SBRT efficacy. STAR VT (NCT04612140), Radiate VT (NCT05765175) and Radioablate VT (TBD), led by our centre, are three randomised controlled trials which will look to compare SBRT to CA outcomes in patients who have failed CA. CARA VT (NCT05047198) is another randomised controlled trial which will look to compare SBRT to CA outcomes in patients who have failed CA or who have VT requiring intervention with a PAINESD score ≥ 15. In the CARA VT trial, ECGi will be leveraged in the SBRT arm to allow completely non‐invasive electrical mapping of substrate and treatment of patients.

## Conclusions

SBRT is a novel, attractive treatment modality for VA that can be achieved with a completely non‐invasive approach sparing patients from the risks of invasive procedures and general anaesthetic which are often poorly tolerated. The short duration of therapy improves the tolerability of the procedure especially in those with concomitant heart failure and frailty. Future mechanistic and clinical studies will be essential to improve long‐term efficacy and reduce the risk of adverse effects.

## Conflict of interest

Verity Ann Ahern is an Editorial Board member of JMIRO and a co‐author of this article. To minimise bias, they were excluded from all editorial decision‐making related to the acceptance of this article for publication.

## Data Availability

Data sharing is not applicable to this article as no new data were created or analyzed in this study.
